# Atlanta Society of Medicine

**Published:** 1886-01

**Authors:** 


### Society Reports.

### ATLANTA SOCIETY OF MEDICINE.

Reported for THE ATLANTA MEDICAL AND SURGICAL JOURNAL.

STATED MEETING, November 3, 1885.

President J. D. Wilson in the chair.

Dr. Elkin reported a case of syphilis in which the initial lesion was situated in the urethra. The patient, a negro boy, consulted him September 24th, 1884, concerning a slight gleety discharge from the urethra, accompanied by more or less pain on urinating. These symptoms had continued quite two months, during which time the boy had used various astringent injections, together with internal remedies, for the purpose of curing what he supposed to be a clap. Dr. Elkin did not make a careful examination on the first visit, but supposing the case to be one of chronic gonorrhœa, the treatment was directed to that end. As the symptoms did not subside after several days' treatment, he made a more thorough investigation into the nature of the case, and by the introduction of bulbous pointed sounds in the urethra and palpation along its floor he was enabled to locate a circumscribed induration about the size of a small pea, two inches within the meatus urinarius. He found the chain of glands in the groins enlarged, as were the post-cervical and epitrochlear, proving more conclusively the specific character of the disease. The next morning after this examination the secondary manifestations had made their appearance and the patient was directed to take pills of blue mass and iron. In the course of a few weeks the discharge from the urethra ceased, the induration of the initial lesion disappeared and the secondary manifestations gradually faded away.

This case was reported, not on account of any peculiarity in the nature of the disease or in the form of treatment employed, but on account of the position of the initial lesion in the urethra, which caused it to resemble so closely chronic gonorrhœa and gleet.

Dr. Virgil O. Hardon reported

A CASE OF EPITHELIOMA OF THE CLITORIS.

A. D., white, married, aged 56, ceased to menstruate twelve years ago after having born ten children. Her general health has always been good. No family history of cancer. Ten months ago she began to notice a soreness on the clitoris, which was soon followed by ulceration. This has slowly spread up to the present time. Examination showed an ulcer about the size of a dime at the site of the clitoris and extending laterally upon the labia. The clitoris was entirely destroyed. This ulcer was surrounded by indurated tissue for about a quarter of an inch in all directions. There was no extension of the disease to the uterus, vagina, bladder or urethra and no tenderness or enlargement of the inguinal glands.

October 15.—Patient was etherized, and all the diseased tissue, making a mass about the size of an English walnut, was removed with the scissors. The labia were brought together by wire sutures passed under the whole wound as in perineorraphy. Union by first intention took place throughout. A portion of the tissue removed was subjected to microscopic examination and found to contain the concentric nests of epithelial cells, the pearly bodies characteristic of epithelioma.

Epithelioma of the external female genital organs is not common. Few of the text-books make any mention of it. Jonathan Hutchinson, in writing on the subject, was able to collect but fourteen cases. In all these the labia were the parts affected, and in two cases the clitoris and nymphæ also. The longest time the disease had existed in any case was five years. It returned after operation in three of the cases. Operation is said to have been finally followed by recovery in the other cases, save one, where the result is not given.

Also,

A CASE OF LACERATION OF THE CERVIX UTERI, in which trachelorraphy was performed three times before a successful result was obtained.

The patient, a widow, white, aged 36, has born seven children. Her health has always been good until three years ago, when she began to suffer from uterine symptoms accompanied by general deterioration of health. She consulted me in the spring of 1883, when I detected bilateral laceration of the cervix, and found a considerable amount of cicatricial tissue on both sides. As I was about to leave the city I was unable to take charge of the case. The following autumn she went to New York and entered the Woman's Hospital in that city. Trachelorraphy was performed twice within an interval of two months and both operations failed, the lips uniting only partially. She returned to this city without any amelioration of her symptoms. Last July she again came under my care. I found the laceration partially closed, and the same cicatricial condition of the cervix, but to an increased degree. After a course of preparatory treatment on the 18th of October, I operated upon this patient, removing from each side of the cervix a V-shaped piece, including the laceration and all the cicatricial tissue that could be found. The lips were then brought together by wire sutures after Emmet's method. Nine days later the sutures were removed and union by first intention was found to have taken place, so that the cervix was restored very nearly to the appearance and condition of that of a nullipara.

The interesting point in this case lies in the fact that the third operation was successful after two futile attempts had been made. I attribute the failure in the first two operations to the fact, which was evident, that in those operations the cicatrices involved in the laceration were not removed, but the effort was made to unite surfaces which were composed of cicatricial tissue. The injurious effects of cicatricial tissue in the cervix in the induction of reflex neuroses by pressure upon nerve filaments are now well understood, but I think that attention has not been sufficiently drawn to the additional fact that it is difficult, if not impossible, to obtain a permanent and satisfactory union of the denuded surfaces in the operation of trachelorraphy when such tissue is allowed to remain in the wound.

Dr. N. O. Harris reported a case in which the injection of quinine produced a herpetic eruption of the glans penis. The patient was twenty-eight years old, unmarried, and of temperate habits. He had been frequently treated by Dr. Harris for slight indispositions, for which he had occasion to prescribe quinine, with the above result. Suspecting that the quinine was the cause of this vesicular eruption, the dose of the drug was diminished, but the herpes still occurred. The same patient, even when taking the smallest doses of quinine, would suffer from the same characteristic eruptions between the fingers and toes.

Dr. Wile reported

### A CASE OF MILIARIA.

A young man 28 years of age was referred to me a few days ago, who was terribly distressed about an eruption which appeared upon his skin, and which had then existed already two weeks. The cause of his anxiety was the fact that six months ago he had a lesion on his penis. This lesion had been pronounced specific by a physician, and the patient was told to look out for cutaneous manifestations. His search was rewarded, for an eruption made its appearance. The patient is a well nourished, rather corpulent individual, and he exhibited an acute inflammatory eruption, characterized by pin-point to pin-head sized vesicles situated on a bright red inflammatory base.

The disease is known as miliaria, or prickly heat, and in this case was scattered over the trunk and extremities, but most marked on the sides of the trunk, especially the abdomen. In this location, large, diffuse, bright red patches could be seen thickly studded with vesicles. Some of the vesicles were on the decline—drying up ; others were developing, it being the nature of the disease to appear in successive crops. It may last a few days or an entire season, if the patient is negligent, and under the latter condition, on account of the burning and tingling, which obliges the patient to scratch more or less, dermatitis or eczema may supervene. The disease is caused by excessive heat and heavy underclothing, giving rise to profuse perspiration, as in this case, where the patient works hard and perspires freely and continuously. It is an affection so marked that it is almost impossible to be confounded with any other cutaneous eruption, especially syphilis. Its appearance during hot weather, usually on corpulent individuals who perspire freely, the burning and tingling sensation and the sudden disappearance under treatment, is sufficient to distinguish it clearly.

The treatment is simple and satisfactory, and in this case consisted in the use of an astringent lotion of zinc sulphate, one drachm to water one pint, followed by an absorbent powder of oxide of zinc and starch.

Dr. Wilson reported

A CASE OF VIRULENT BUBO IN THE FEMALE.

Both groins were affected. The abscesses had burst spontaneously, but the doctor did not see the case until a considerable time after this event. When seen, there was a furrow on either side large enough to accommodate one or two fingers of an adult, and the phagedenic and burrowing process was still acutely progressing. In either groin was simply a huge phagedenic chancroid. On the one side there was but one opening, large and patulous; on the other, in addition to the principal opening, were one or two smaller fistulas communicating with the main excavation. No history of chancroid could be made out. The patient had gonorrhœa at the time, but as a virulent bubo cannot result from gonorrhœa, the pre-existence of chancroid is practically certain. With the assistance of Dr. McRae, the patient had been anæsthetized, and nitric acid had been thoroughly applied. At present, which is some days after the operation, though the furrows have not filled up very much, they are beginning to do so, and they no longer present the corroding and phagedenic appearance. They are kept well cleansed with carbolized water, and the only dressing is iodoform and absorbent cotton. Iron and quinia are used internally. The reporter saw the doctor a few days after the meeting, and he says that while the ulcers are rapidly healing, they are doing so by a kind of adhesive inflammation, which will leave a large hardened ridge in either groin.

