# A Correction

**Published:** 1886-01

**Authors:** 


A CORRECTION.—On page 671 of this JOURNAL, fifth line, injestion should be read for injection.

